# One-step artificial antigen presenting cell-based vaccines induce potent effector CD8 T cell responses

**DOI:** 10.1038/s41598-019-55286-5

**Published:** 2019-12-12

**Authors:** Qingtai Su, Botond Z. Igyártó

**Affiliations:** Baylor Scott & White Research Institute, Baylor Institute for Immunology Research, Dallas, TX USA

**Keywords:** Cancer immunotherapy, Immunization

## Abstract

The production and wide use of artificial antigen presenting cells (aAPCs) in the clinic as cancer immunotherapeutics are hindered by the need of identifying immunogenic cancer antigens and production of recombinant patient-specific major histocompatibility complexes (MHC) loaded with these peptides. To overcome these limitations, in this study, we tested the idea of whether peptide-MHCs can directly be captured from cell lysates, including cancer cells using affinity beads, and used to initiate T cell responses. In theory, these affinity beads covered with the unknown peptide-MHC repertoire captured from the cancer cells could interact with a wide range of antigen-specific T cells and promote anti-cancer responses. Indeed, we found that we can successfully pull-down peptide-MHCs from cell lysates and the aAPCs generated using this technique were able to induce antigen-specific cytotoxic effector T cell responses that led to *in vitro* and *in vivo* tumor cell killing. In summary, we present here a novel technique to generate patient-specific aAPCs, that might have the potential to revolutionize the field of cancer vaccines, and provide patients with a vaccine in matters of days at minimal costs.

## Introduction

Dendritic cells (DCs) as professional antigen presenting cells (APCs), coordinate every aspect of immunity^1^. DCs stimulate and train other immune cells, such as T cells, of the adaptive immune system by presenting peptide epitopes derived from antigens (self, cancer neoantigens, foreign, etc.) on their MHC, and providing co-stimulatory signals in membrane bound and secreted (cytokines) forms^1,2^. These three signals train the T cells to recognize, destroy or tolerate the cells that carry these antigens. Thus, not surprisingly the DCs are the main target of immunotherapies. Two main strategies are used to exploit DCs for cancer therapy^3^. One of them require isolation of patient monocytes from blood and complex *in vitro* manipulation that involves differentiation and maturation into DCs using cytokine and adjuvant cocktails and pulsing with the chosen antigen(s)/cell lysates, followed by reinfusion into the patient. An alternative approach uses antibody as a carrier to deliver antigens to DCs *in vivo*. However, both of these approaches showed only limited success rate, mainly because the DCs’ function is highly dependent on the tumor environment which is often time inhibitory^2,3^.

Artificial antigen presenting cells (aAPCs) could be a viable alternative to live DCs since they do not react to the environmental clues^4–11^. However, present day aAPCs are limited by the fact that researcher conjugate known, genetically engineered and expressed peptide-MHC on beads to stimulate T cell responses – a method limited to defined and selected MHC/peptide complexes. The need of prior identification of immunogenic tumor neoantigens and *in vitro* synthetization of corresponding patient-specific MHC-I haplotype has limited the widespread use of aAPCs in the clinic^12,13^.

Here we present an alternative approach to generate aAPC-based cancer vaccines that does not require identification and *in vitro* production of the peptide-MHCs. This is a one-step process that allows the capture of the peptide-MHCs directly from the patient-derived tumor cell lysates to generate aAPCs. We bring experimental evidence that peptide-MHC-I repertoire of normal- or tumor cells can be successfully captured directly from cell lysate using affinity beads. The aAPCs generated using this technique were able to induce antigen-specific cytotoxic effector T cell responses that led to *in vitro* and *in vivo* tumor cell killing. Collectively, our novel aAPCs production strategy show potential in revolutionizing aAPC-based cancer immunotherapy.

## Materials and Methods

### Mice

OT-I Rag2^−/−^ CD8 TCR transgenic mice specific for OVA_257–264_ (B6.129S6-Rag2tm1Fwa Tg(TcraTcrb)1100Mjb) presented on H-2Kb and WT C57BL/6 mice were purchased from Taconic Biosciences (Rensselaer, NY). All experiments were performed with 8 to 26-week-old female and male mice. Mice were housed in microisolator cages and fed autoclaved food and acidified water. The Baylor Institutional Care and Use Committee approved all mouse protocols. All experiments were performed in accordance with relevant guidelines and regulations.

### Cell lines

B16-OVA (B16F10 tOVA GFP, expressing truncated OVA and GFP) and parental B16F10 are a gift of Drs. Michael Gerner and Andrew Oberst (University of Washington). HEK293T cell line was purchased from ATCC (Manassas, VA). Cells were cultured in Dulbecco’s Modified Eagle Medium (Gibco, Grand Island, NY) supplemented with 10% FBS, 1% Glutamax and 1% sodium pyruvate.

### H-2Kb/OVA expression

The H-2Kb sequence was sub-cloned into cetHS-puro plasmid. As a result, Ctag sequence was fused to the C-terminus of the H-2Kb sequence. The successful generation of the construct was determined by PCR and sequencing (data not shown). One day prior to transfection the HEK293T cells were seeded in 10 cm tissue culture dish. By next day the cells reached 70–80% confluence. At this time, the culture medium was replaced with 9 mL DMEM medium containing 25 μM chloroquine and the cells transfected with plasmids coding for Kb-Ctag and OVA (pcDNA3-OVA; Addgene, plasmid #64599). Briefly, 5 μg of Kb-Ctag and 5 μg OVA expressing plasmids were mixed in 450 μL H_2_O in 1.5 mL Eppendorf tube; 500 μL 2X HBSS was added sequentially. 50 μL 2 M CaCl_2_ solution was then added and the tube was vortexed and kept on ice for 15 minutes. The plasmids were gently added on top of the cell cultures. For single transfections 10 μg of Kb-Ctag plasmid was used. On day 2 post transfection the cells were washed with warm DMEM medium twice and cultured for one extra day.

### aAPC production

Kb-Ctag and OVA expressing 293 T cells (or Kb-Ctag expressing B16F10 cells) were lysed in lysis buffer (1%CHAPS, 25 mM Tris pH 7.5, 150 mM NaCl) containing protease inhibitor (cOmplete ULTRA^TM^ Tablets; Roche, Mannheim, Germany). Lysis was performed at 4 °C for 1 hour. Supernatant was acquired by centrifuging the lysate at 12,000 rpm for 20 minutes. The cleared lysate was then mixed with Ctag matrix (CaptureSelect™ C-tag Affinity Matrix, Thermo Scientific, Waltham, MA) and incubated at 4 °C, on a slowly rotating surface for one hour. The matrix was then washed extensively with sterile PBS (500 rpm/20 seconds spin was used to recover the matrix). The successful pull-down of Kb:SIINFEKL pMHCI complex (or Kb) was determined by staining the matrix with antibodies that detect Kb and/or SIINFEKL bound to H-2Kb.

### *In vitro* OT-I T cell activation

Secondary lymphoid organs from OT-I Rag1^−/−^ mice were smashed through cell strainers and the red blood cells lysed using ACK. After washing, the cells were used as is or labeled with cell trace violet (CellTrace™ Violet Cell Proliferation Kit, Invitrogen, Carlsbad, CA) according to the manufacturer’s instructions. The cells were then seeded in a 24-well plate in complete RPMI medium, each well containing 4 million cells in 2 mL medium. Control and experimental aAPCs (20 μL matrix for one well) were added to the cell cultures for 6 days. Every other day, half of the medium was replaced with fresh medium.

### Flow cytometry

Staining was performed as previously described^14^. Intracellular cytokine staining was performed with the BD Bioscience Cytofix/Cytoperm kit (BD Biosciences, San Jose, CA), according to the manufacturer’s instructions. Samples were analyzed on LSRFortessa flow cytometer (BD Biosciences, San Jose, CA). The fluorochrome-conjugated antibodies to IFN gamma (XMG1.2), granzyme B (QA16A02), H-2Kb bound to SIINFEKL (25-D1.16), CD3ε (145–2C11), CD44 (IM7), CD90.1 (OX-7) and CD8a (3–6.7) were purchased from BioLegend (San Diego, CA). Anti-Kb antibody (Y-3) was purchased from BioXCell (West Lebanon, NH) and conjugated with Alexa Fluor™ 647 antibody labeling Kit (Invitrogen, Carlsbad, CA). Data were analyzed with FlowJo software (TreeStar; Ashland, OR). All the flow cytometric plots displaying cells were pre-gated on live cells using Fixable Viability Dye eFluor 780 (eBioscience, San Diego, CA) and singlet events.

### Adoptive transfer of OT-I T cells into tumor bearing mice

Eight-to-twelve weeks old female and male WT C57BL/6 mice were inoculated subcutaneously with 10^6^ B16-OVA cells. When the tumors became palpable (day 7) each mouse received through tail vein injection 4 × 10^6^ OT-I T cells stimulated *in vitro* with SIINFEKL, control or Kb/OVA aAPCs for 5 days. Female OT-I mice were used as T cell source. Tumors were measured using a caliper at the indicated time points and tumor volumes calculated based on the following formula: volume = (W^2^*L)/2, where W is width and L is length. As per approved animal protocol, the mice in which the tumor size has reached 1,000 mm^3^ or the animals showed distress such as visible weight loss, lack of grooming and feeding were euthanized.

### Statistical analysis

Statistical analyses were performed using GraphPad Prism7.0 software (Graphpad, La Jolla, CA). Statistical methods used to determine significance are listed in the figure legends. A p value < 0.05 was considered statistically significant.

## Results

### The concept behind one-step aAPCs

**Figure 1:**
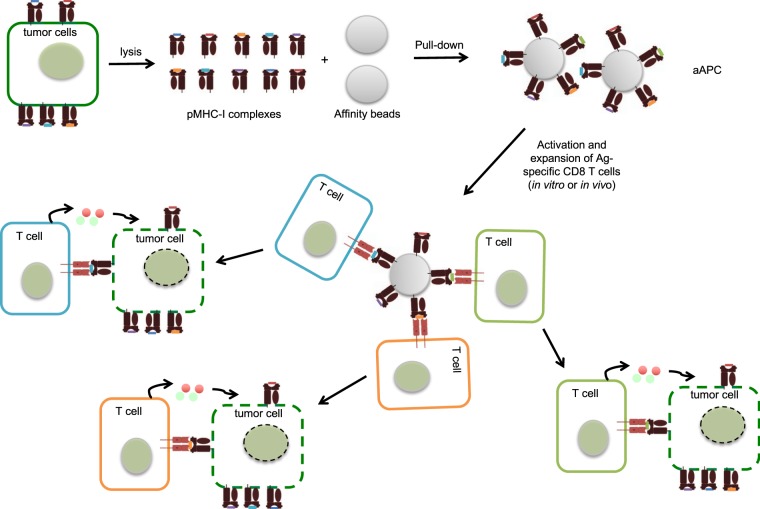
Graphical abstract. Graphical representation of one-step aAPC generation. Tumor cell lysates are incubated with affinity beads that capture the peptide-MHC-I repertoire of the cancer cells. The beads are then used to activate cancer antigen-specific T cell clones that will ultimately kill the tumor cells.

**Figure 2:**
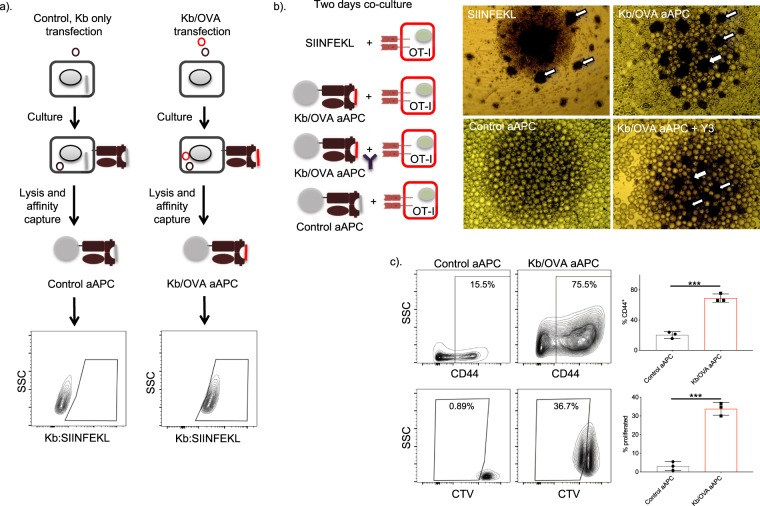
aAPCs loaded with Kb:SIINFEKL can prime OT-I T cells. (**a**) Experimental flow: 293T cells were transfected with either Kb-Ctag (brown) coding plasmid alone or in combination with OVA (red) coding plasmid. After two days of culture the cells were lysed and the MHC-Is captured using affinity beads targeting the Ctag motif. The capture of MHC-I complexed with SIINFEKL were determined with the help of flow cytometry using antibody that recognizes SIINFEKL bound to Kb. One representative experiment out of three is shown. **(b**) OT-I cells were stimulated *in vitro* with SIINFEKL peptide, Kb/OVA aAPCs, control aAPCs or Kb/OVA aAPCs in the presence of Y3 antibody, and pictures taken two days later (right). Arrows point to some of the activated OT-I cell clusters induced by the stimulation. One representative experiment out of three is shown. Three samples/group, except SIINFEKL that served the role of positive control. (**c)** In a separate experiment, OT-I cells were stimulated with either Kb/OVA aAPCs or control aAPCs for 2 days and assessed by flow cytometer for activation (CD44) and proliferation (CTV dilution). One representative experiment out of three is shown, n = 3. Unpaired t-test. ***p < 0.001.

The production and wide use of aAPCs as cancer immunotherapeutics is hindered by the need of identifying immunogenic cancer antigens and production of recombinant patient-specific HLAs loaded with these peptides. To overcome these limitations, we tested the idea whether peptide-MHCs can directly be captured from cell lysates, including cancer cells using affinity beads. In theory, these affinity beads covered with the unknown peptide-MHC repertoire captured from cancer cells could interact with a wide range of antigen-specific T cells and promote anti-cancer responses. The concept and strategy to generate one-step aAPCs are depicted in Fig. 1/Graphical abstract. For the proof of concept experiment we designed a system where we can control and confirm each step of the aAPC generation. As a first step we transfected human embryonic kidney cells (HEK293T) with plasmids coding for C-tagged mouse H-2Kb (MHC-I) and OVA (Fig. 2a). The expression of the mouse H-2Kb on the cells surface was detected by Y-3 antibody (recognizes H-2Kb) and the formation of OVA-derived peptide MHC complexes with the use of antibody that recognizes SIINFEKL (dominant, OVA-derived CD8 epitope) bound to H-2Kb. We observed efficient expression of H-2Kb and loading of SIINFEKL peptide on MHC-I molecules (data not shown). No SIINFEKL staining was observed in the cells transfected only with Kb-Ctag plasmid (data not shown). After confirming the expression of OVA-derived peptide in the context of MHC-I, we lysed the cells and purified the peptide-MHC-Is using affinity matrix targeting the C-tag motif of the H-2Kb. The capture of MHC-I and MHC-I complexed with SIINFEKL were determined with the use of antibodies already presented above. We found that the affinity beads efficiently captured MHC-I and MHC-I loaded with SIINFEKL (Fig. 2a). Thus, these data suggested that affinity beads targeting MHC-I could be an easy way to generate patient-specific aAPCs.

### aAPCs loaded with Kb:SIINFEKL can prime OT-I T cells

The successful generation of aAPCs using affinity matrix prompted us to test whether the generated aAPCs can activate antigen-specific CD8 T cells. For this purpose, we generated aAPCs as presented above and co-cultured them with naïve OT-I cells. OT-I cells recognize SIINFEKL peptides presented in the context of H-2Kb. SIINFEKL peptide (2 μg/mL) stimulation served as a positive control (Pulle *et al*., 2006). The *ex vivo* culturing of OT-I T cells with aAPCs carrying Kb:SIINFEKL (Kb/OVA), but not control aAPCs (Kb), led to cell cluster formation (Fig. 2b). Inclusion of Y-3 antibody in cultures that binds to peptide groove of the H-2Kb, and thus interfere with TCR binding, decreased OT-I cluster formation (Fig. 2b). To confirm the activation and proliferation of OT-I cells, CTV-labeled OT-I cells were co-cultured with either control or Kb/OVA aAPCs and analyzed by flow cytometer. Kb/OVA aAPCs, unlike control aAPC, induced significant upregulation of CD44 and dilution of the proliferation dye by day 2 (Fig. 2c). AAPCs generated using the same technique but a different cell line (B16F10) provided similar results (data not shown). Together, these data suggest that our aAPCs were able to prime antigen-specific T cell responses.

### aAPC-activated T cells can kill tumor cells *in vitro* and *in vivo*

**Figure 3:**
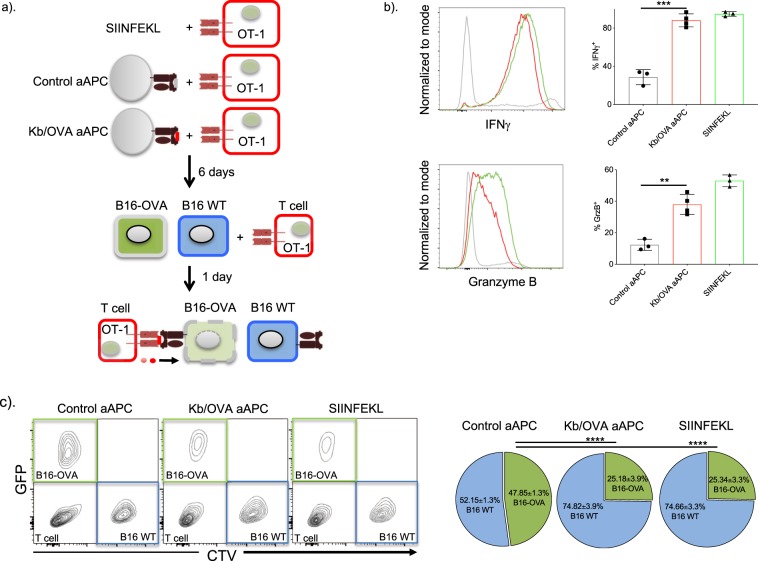
aAPC-activated OT-I T cells can kill tumor cells *in vitro*. **(a)** Experimental flow. OT-I cells were stimulated with SIINFEKL, control or Kb/OVA aAPCs for 6 days. **(b**) Before their use for experiments the OT-I T cells’ cytotoxic phenotype was confirmed by flow cytometry. Unpaired t-test, **p < 0.01, ***p < 0.001. (**c**) The OT-I cells were then mixed with B16-OVA (target cells; green) and B16 WT (control cells; blue) cells and cultured for one day. The killing of target cells was determined by flow cytometer (left) and shown as relative percentage (right). One representative experiment out of two is shown, n = 3. One-way ANOVA on green pie segments, ****p < 0.0001.

**Figure 4:**
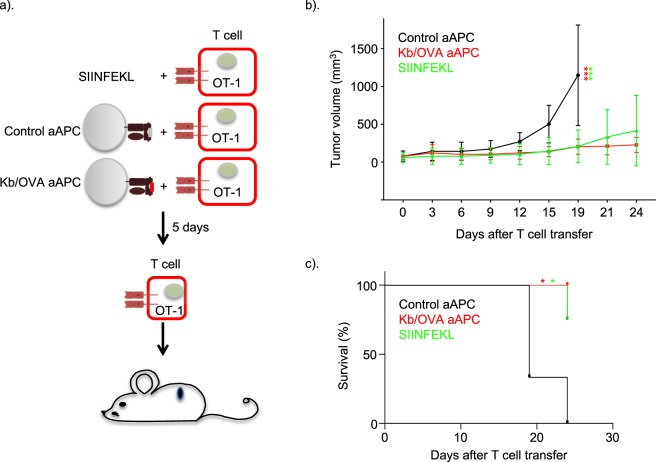
aAPC-activated OT-I T cells can kill tumor cells *in vivo*. (**a)** Experimental flow. OT-I cells were stimulated with SIINFEKL, control or Kb/OVA aAPCs for 5 days. To determine the *in vivo* tumor killing potency of the aAPC-stimulated OT-I cells, the OT-I cells were transferred into B16-OVA tumor bearing mice and the tumor growth (**b**) (two-way ANOVA; Tukey’s test ***p < 0.001) and the survival monitored as depicted (**c**) (Long-rank, Mantel-Cox test, *p < 0.05). Data from two experiments were combined. N = 6–8 mice/group.

To investigate the cytotoxic potency of the aAPC-primed T cells, we designed an *in vitro* tumor cell-killing assay using the B16F10 cell line. OT-I T cells were first primed by Kb/OVA aAPCs or control aAPCs for 6 days *in vitro* (Fig. 3a). Prior use the cytotoxic phenotype (IFNγ and granzyme B) of the primed OT-I cells were confirmed by flow cytometry (Fig. 3b). B16F10 cells that express OVA and GFP (hereafter B16-OVA) were then mixed with their parental B16F10 cells (B16 WT) that were labeled with CTV at a ratio of 1:1 (Fig. 3a). The primed OT-I T cells were added directly to B16 cell cultures one day after seeding at an effector to tumor cell ratio of 2:1. Peptide-stimulated OT-I cells served as positive controls. Cell counts were read by FACS one day after T cell addition. Both peptide- and aAPC-stimulated T cells effectively killed the tumor cells expressing OVA, but left the parental WT tumor cells intact (Fig. 3c). To further interrogate the cytotoxic ability of aAPC-stimulated T cells, we infused the OT-I T cells intravenously into WT mice carrying B16-OVA tumors (Fig. 4a). Both peptide and aAPC-stimulated OT-I cells significantly slowed the tumor growth (Fig. 4b) and increased survival (Fig. 4c). Collectively, our data suggested that this novel aAPC was able to induce cytotoxic T cells and that could potentially be used as immunotherapeutic.

### aAPCs generated using tumor cells were able to activate T cells from tumor-bearing mice

**Figure 5:**
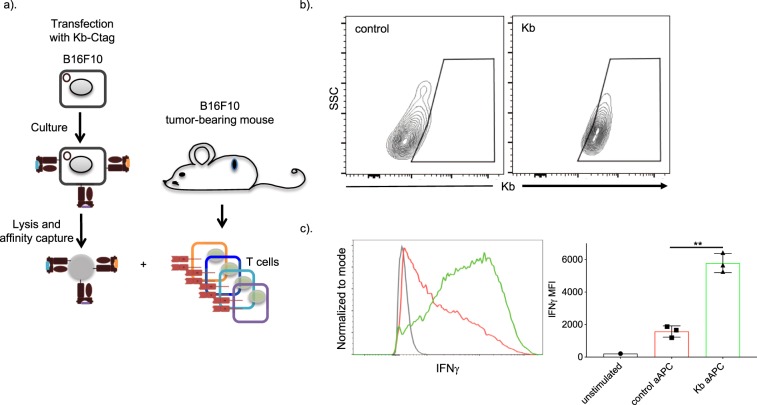
aAPCs generated using B16F10 cells activated T cells from tumor-bearing mice. (**a**) Experimental flow for data presented on figures **(b,c**). B16F10 cells were transfected with Kb-Ctag plasmid. Two days later the cells were lysed and the MHC-I repertoire captured using affinity beads. The successful capture of the MHC-Is was confirmed with flow cytometry (**b**). The aAPCs were then used to stimulate T cells isolated from tumor bearing mice’s spleen for six days. The induction of CD8 T cells with cytotoxic phenotype, characterized by IFNγ production, was confirmed using flow cytometry (**c**). One representative experiment out of two is shown, n = 3, except unstimulated n = 1. Unpaired t-test. **p < 0.01.

The success with the model antigen OVA, encouraged us to explore whether aAPCs generated by using unknown tumor antigens could be used to stimulate T cells isolated from tumor-bearing mice. To address this question, we first transfected B16F10 cells with C-tagged H-2Kb and isolated the peptide-MHC-Is as presented above. The scientific rational behind this experiment was that these MHC-I molecules will be loaded with tumor antigens and we can then capture the peptide-MHC-I repertoire of the B16F10 cells using affinity beads (Fig. 5a). FACS assay confirmed the successful capture of H-2Kbs from C-tagged H-2Kb transfected B16F10 cells (Fig. 5b). As next, splenocytes isolated from B16F10 tumor-bearing mice were isolated and stimulated with experimental and control aAPCs. We observed that our experimental aAPCs, comparing to control aAPCs activated significantly higher numbers of CD8 T cells to produce IFNγ (Fig. 5c). Thus, the data support that generation of patient-specific aAPCs is plausible.

## Discussion

Here we present a novel and simple technique to generate patient-specific cancer vaccines. We bring experimental evidence that can serve as the basis of the generation of patient-specific vaccines targeting cancer and autoimmune diseases. We show that affinity beads can be used to pull down peptide-MHC directly from cell lysates and it is a viable option to generate patient-specific aAPCs in matters of days at minimal costs.

The clinical usability of aAPCs generated until now to treat cancer were limited by multiple factors. AAPCs have to be patients specific that first require characterization of the patient’s HLA haplotype and identification of cancer neoantigens. The HLA haplotype is fairly easily determined by PCR and flow cytometry, however identification of immunogenic cancer neoantigens is a very expensive, tedious, and labor-expensive process with many caveats along the pipeline that further limit its success rate^15–18^. Since most of the cancers are highly heterogeneous a dominant neoantigen might only target some of the cancer cells, and the neoantigen could be patient-specific or it will only bind to certain HLA molecules, limiting their wider usability. To make the aAPCs, the correct HLAs have to be produced as recombinant proteins and assembled with the corresponding neoantigenic peptides. The recombinant HLA and peptides might not carry all the posttranslational modifications that would normally occur *in vivo* that could result in less effective TCR stimulation. Furthermore, the whole spectrum of HLA is hard to reconstruct. To simplify the generation of aAPCs, we decided to focus on things that are important and disregard the need to identify antigens. We hypothesized that we could generate aAPCs by simply pulling down peptide-MHCs directly from tumor lysates. Indeed, we found that we can successfully pull-down peptide-MHCs that can later be used to stimulate antigen-specific anti-cancer effector T cell responses. Tumor-specific T cell clones are of low abundance in nature^19^, and unlike most of the aAPCs generated to date, which often only present one antigenic peptide, this technique, by capturing a diverse peptide-MHC-I pool should increase the chance to target and activate multiple cancer-specific T cell clones. It is expected that the peptide repertoire presented by the cancer cells and the tumor heterogeneity will be represented proportionally on the aAPCs. If later studies will prove it otherwise, we can use a modified version of the Drop-sequencing technology, where in this case a lipid droplet containing lysis buffer will form around one cancer cell and one affinity bead. This will assure that every aAPC will represent one cancer cell’s peptide-MHC-I repertoire. Tumor samples with higher leukocytic infiltrates might require purification steps to enrich for tumor-derived signatures. In some instances tumor cells escape immune surveillance by downregulating surface expression of MHC-I^20^. In theory, our aAPCs could still capture the intracellularly retained peptide-MHC-I repertoire; or as a last resort, ectopic transfection of tumor cells with patient-specific HLA-I could circumvent this caveat. The aAPCs generated using this technique will probably contain, self, non-mutated, non-immunogenic peptides that could trigger some sorts of autoimmune responses if combined with co-stimulation. This, however, is expected to be minimal, because of central and peripheral tolerance. The possible autoimmune symptoms could also be controlled by the use of different drugs.

Because of lack of access to antibodies that would recognize the cytoplasmic part of mouse H-2Kb and for versatility reasons, in these proof-of-concept *in vitro* and *in vivo* experiments, we used tag-specific antibodies. Future studies should consider antibodies that recognize cytoplasmic portion of the Kb or target beta-2 microglobulin to capture the peptide-MHC-I complexes from C57BL/6 mouse samples. For human studies and for the generation of human aAPCs the W6/32 pan HLA-I antibody could prove to be a good candidate. Viral proteins that interact with the cytoplasmic portion of HLA-I could be also a viable option for affinity purification of peptide-MHC-I repertoire and generation of aAPCs.

In theory this technique can be applied to achieve both immunogenic and tolerogenic immune responses. AAPCs generated from normal cells or cells pulsed with self-antigen and combined with inhibitory signals and cytokines should generate antigen-specific regulatory T cell responses (prevent or treat autoimmune diseases). Along the same logic aAPCs could be generated to treat allergy or used as preventative vaccines to fight infectious diseases.

In summary, here we presented the scientific community with a concept that has the potential to revolutionize cancer vaccines and make it affordable to anybody in matter of days giving a chance also to the patients with very limited time on their hands. Of course, more research will be needed to perfect this technology, including but not limited to carrier’s characteristics (material, size, shape, *in vivo* half-life, controlled release etc.), co-stimulatory molecules, cytokines and chemokines to use, route of delivery etc.

## Data Availability

Datasets are included with the manuscript.
